# Supplementary material for: Association between oral microbiome and seven types of cancers in East Asian population: a two-sample Mendelian randomization analysis
Source: Front Mol Biosci. 2023 Nov 21;10:1327893. doi: 10.3389/fmolb.2023.1327893 (PMC10702768; doi:10.3389/fmolb.2023.1327893)
Supplement: Supplementary file 1 [file DataSheet1.ZIP › Supplementary Materials/MR plots of saliva/saliva═╝/Prostate cancer/pheno.3242_to_prostate cancer_leave_one_out.pdf]

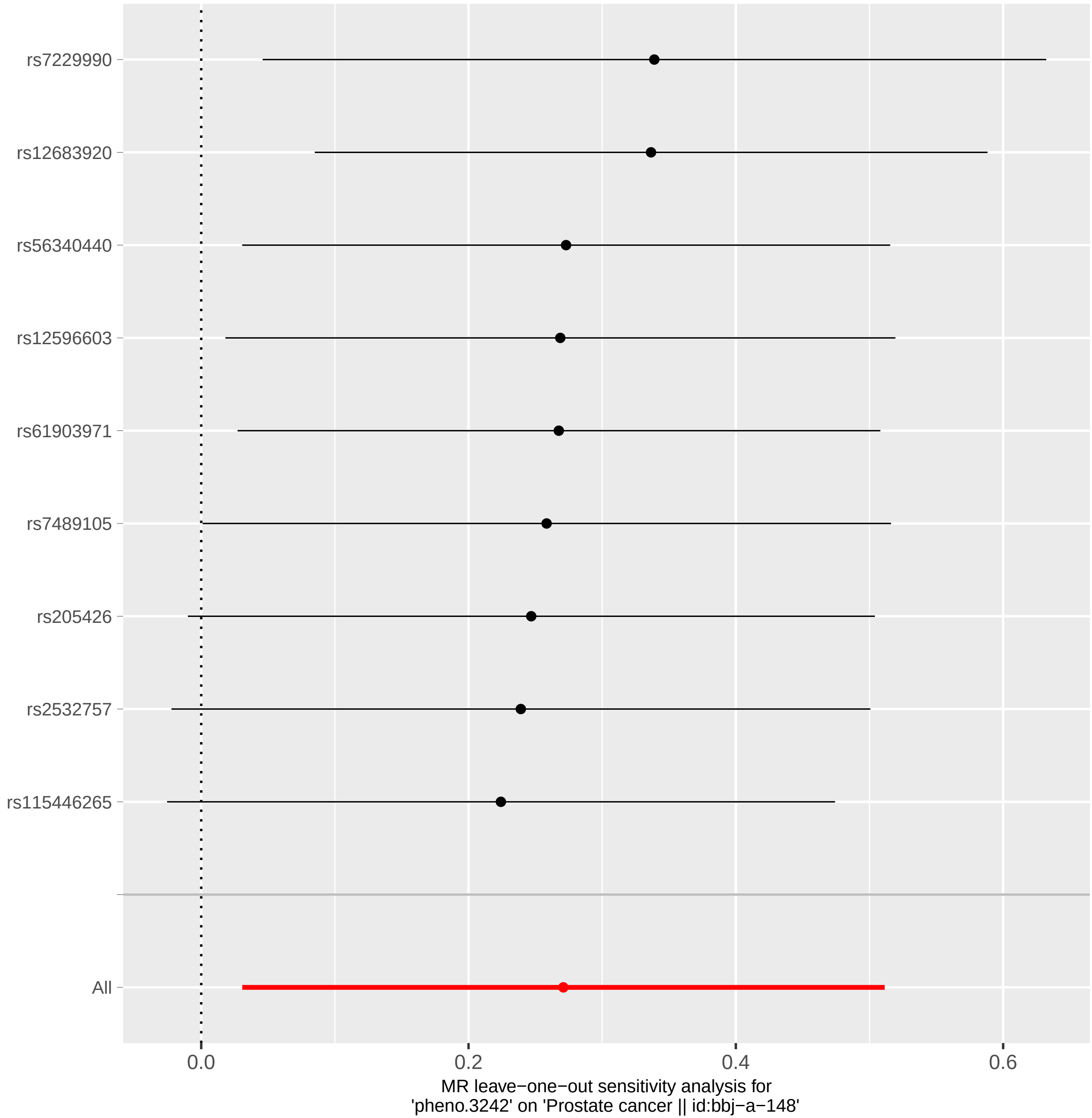
rs7229990
rs12683920
rs56340440
rs12596603
rs61903971
rs7489105
rs205426
rs2532757
rs115446265
All
0.0
0.2
0.4
0.6
MR leave−one−out sensitivity analysis for
'pheno.3242' on 'Prostate cancer || id:bbj−a−148'
